# Time-Programmed Delivery of Sorafenib and Anti-CD47 Antibody via a Double-Layer-Gel Matrix for Postsurgical Treatment of Breast Cancer

**DOI:** 10.1007/s40820-021-00647-x

**Published:** 2021-06-12

**Authors:** Liping Huang, Yiyi Zhang, Yanan Li, Fanling Meng, Hongyu Li, Huimin Zhang, Jiasheng Tu, Chunmeng Sun, Liang Luo

**Affiliations:** 1 College of Life Science and Technology, National Engineering Research Center for Nanomedicine, Huazhong University of Science and Technology, Wuhan, 430074 People’s Republic of China; 2 State Key Laboratory of Natural Medicines, NMPA Key Laboratory for Research and Evaluation of Pharmaceutical Preparations and Excipients, and Department of Pharmaceutics, School of Pharmacy, China Pharmaceutical University, 24 Tong Jia Xiang, Nanjing, 210009 China; 3 Hubei Key Laboratory of Bioinorganic Chemistry and Materia Medica, School of Chemistry and Chemical Engineering, Huazhong University of Science and Technology, Wuhan, 430074 People’s Republic of China; 4 School of Food Science and Pharmaceutical Engineering, Nanjing Normal University, Nanjing, 210023 People’s Republic of China

**Keywords:** Hierarchical hydrogel, Sorafenib, Postoperative immunosuppression reversal, Tumor-associated macrophages, Anti-CD47 antibody

## Abstract

The highly immunosuppressive microenvironment after surgery has a crucial impact on the recurrence and metastasis in breast cancer patients. Programmable delivery of immunotherapy-involving combinations through a single drug delivery system is highly promising, yet greatly challenging, to reverse postoperative immunosuppression. Here, an injectable hierarchical gel matrix, composed of dual lipid gel (DLG) layers with different soybean phosphatidylcholine/glycerol dioleate mass ratios, was developed to achieve the time-programmed sequential delivery of combined cancer immunotherapy. The outer layer of the DLG matrix was thermally responsive and loaded with sorafenib-adsorbed graphene oxide (GO) nanoparticles. GO under manually controlled near-infrared irradiation generated mild heat and provoked the release of sorafenib first to reeducate tumor-associated macrophages (TAMs) and promote an immunogenic tumor microenvironment. The inner layer, loaded with anti-CD47 antibody (aCD47), could maintain the gel state for a much longer time, enabling the sustained release of aCD47 afterward to block the CD47-signal regulatory protein α (SIRPα) pathway for a long-term antitumor effect. In vivo studies on 4T1 tumor-bearing mouse model demonstrated that the DLG-based strategy efficiently prevented tumor recurrence and metastasis by locally reversing the immunosuppression and synergistically blocking the CD47-dependent immune escape, thereby boosting the systemic immune responses.

**Supplementary Information:**

The online version contains supplementary material available at 10.1007/s40820-021-00647-x.

## Highlights

- A novel injectable double-layer-gel matrix is developed.
- The hierarchically structured dual lipid gel matrix enables time-programmed drug delivery.
- Applying a modest dose of sorafenib in advance can reeducate tumor-associated macrophages and potentiate anti-CD47 treatment.

**Supplementary Information:**

The online version contains supplementary material available at 10.1007/s40820-021-00647-x.

## Graphical abstract

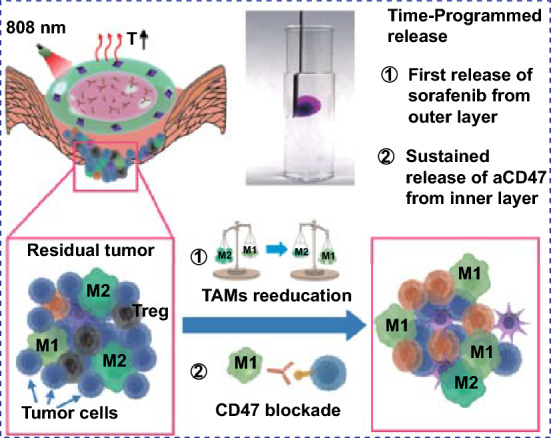

## Introduction

Surgery is among the first-line treatment modalities for solid tumors in clinic [1–3]. However, there is quite a possibility that surgery shows progress for a period of time followed by a stalling or continued growth and metastasis of cancer. Among solid tumors, breast cancer has a particularly high rate of recurrence and distant metastasis due to the inherent invasive ability of tumor cells and rapid vascularization [4]. In addition, the immunosuppression associated with postsurgical wound healing not only promotes cancer cell invasion and proliferation, but also restrains the activity of antitumor leukocytes [5–7]. This immunosuppression is also one of the key factors that obstructs current gold-standard postsurgical cancer treatment approaches, such as chemotherapy and radiotherapy, from achieving desirable clinical outcomes [8–10]. Although cancer immunotherapy has been considered to inhibit tumor recurrence and metastasis, many of these approaches become unfavorable when facing the highly immunosuppressive microenvironment of cancers after surgical treatment [5, 11–16]. Strategies that can reverse postoperative immunosuppression and promote immunogenic tumor phenotype are immediately required to endow desired clinical benefit [17, 18].

Alternatively polarized tumor-associated macrophages (TAMs), or the M2-like TAMs, are prone to accumulating to high levels in postsurgical microenvironment, which is responsible for expediting the malignant tumor cells proliferation and neo-angiogenesis, and further facilitating the progression of them toward a metastasis phenotype [19, 20]. In contrast, classically polarized macrophages, or the M1-like TAMs, can secret a number of proinflammatory cytokines and reprogram tumor cells into an immunogenic phenotype [21–23]. Owing to the plasticity of macrophages [24], reeducating tumor-promoting M2-like TAMs to the tumoricidal, M1-like phenotype denotes an effective strategy to reverse the immunosuppressive microenvironment in postsurgical cancer treatment. Sorafenib, a small molecule multi-kinase inhibitor approved for the treatment of hepatocellular carcinoma, renal cell carcinoma, and others [25], has been reported to modulate macrophage polarization and affect macrophages outside the primary tumor involved in metastasis formation [26, 27], in a dose-dependent manner, thereby representing a promising candidate to alter the function of M2-like TAMs and reverse the immunosuppressive cytokine profile of TAMs.

On the other hand, macrophages are critical mediators of innate immunity and responsible for directly presenting phagocytized foreign substance to T cells [28, 29]. However, a variety of tumor cells have upregulated CD47 protein on their surface, which can interact with signal regulatory protein alpha (SIRPα) on M1-like TAMs and trigger evasion of tumor cells from macrophage recognition [30, 31]. Blocking the interaction of CD47 with SIRPα is able to activate phagocytic cells, including M1-like TAMs and dendritic cells (DCs), and increase tumor cells phagocytosis [32–34]. Moreover, effector T cells can be activated for enhanced antitumor efficacy upon phagocytosis of tumor cells through CD47 blockade [33, 34]. Therefore, combining TAM modulation with CD47-blockade immunotherapy holds great promise for effective prevention of postsurgical tumor recurrence and metastases in clinic. Taking this into account, we hypothesize that sequentially delivering a modest dose of sorafenib prior to CD47-blockade immunotherapy is a rational implementation strategy. By this means, TAMs at the tumor resection sites can be reeducated by sorafenib first, followed by overcoming tumor immune escape via CD47 blockade, thereby establishing an overall immune-favorable microenvironment for enhanced therapeutic outcomes. In addition, from recent clinical trials, CD47 antagonists administered intravenously could cause serious clinical hematotoxicity, such as anemia and thrombocytopenia [35–37], so that it is pivotal to develop a localized delivery matrix that can co-load sorafenib and CD47 antagonist in an “all-in-one” manner and deliver them in a spatiotemporally regulated pattern [38, 39].

**Fig. 1:**
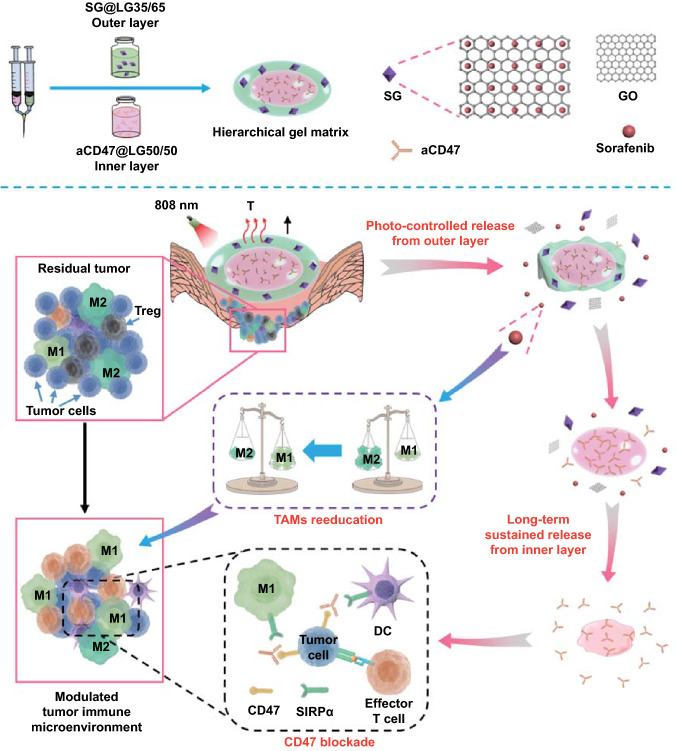
Schematic illustration of time-programmed sequential delivery of combined cancer immunotherapy via a hierarchically structured gel matrix (DLG scaffold) for postsurgical tumor immune microenvironment modulation

In this study, we designed an injectable hierarchically structured gel matrix with dual lipid gel (DLG) layers, the outer and inner layers of which were composed of different mass ratios of soybean phosphatidylcholine (SPC) and glycerol dioleate (GDO), to realize the aforementioned sequential delivery of combined cancer immunotherapy (Fig. 1). We have previously demonstrated that the SPC/GDO binary lipid system is biocompatible and ideal for tunable drug delivery, whose gelation behaviors and drug release profiles can be regulated by simply adjusting the mass ratio of the two lipids [9, 40]. Here, the binary SPC/GDO system with a mass ratio of 35/65 is chosen as the precursor of the outer layer lipid gel (LG) of the DLG matrix, and loaded with sorafenib-adsorbed graphene oxide (GO) nanoparticles (SG). Once injected, the outer layer LG precursor hydrates promptly into a thermal-responsive gel depot that undergoes reversible gel-to-sol transition in response to a small temperature change at around 39 °C [9]. GO under manually controlled near-infrared (NIR, 808 nm) light irradiation can generate mild heat and induce the gel-to-sol phase change of the outer layer LG, and provoke the photo-controlled release of sorafenib to reeducate TAMs and promote an immunogenic microenvironment. The inner layer LG precursor of the DLG matrix, loaded with anti-CD47 antibody (aCD47), is composed of SPC and GDO with a mass ratio of 50/50, and has a much higher phase transition temperature of 92 °C (Fig. S1). It will hydrate at a later stage and maintain the gel state for a much longer time, enabling the sustained release of aCD47 afterward to block the CD47-SIRPα pathway for a long-term antitumor effect. In vivo studies using mouse models bearing 4T1 breast cancer cells, with a relatively insufficient immunogenicity and high tendency to metastasis [38], have validated the time-programmed sequential delivery of sorafenib and aCD47 via this hierarchical structured hydrogel, which successfully modulated the population ratio of M1/M2 and rebuilt an immune-favorable microenvironment. The strategic combined cancer immunotherapy efficiently prevented postoperative tumor recurrence and metastasis by synergistically reversing the local immunosuppression and boosting the systemic immune responses.

## Results and Discussion

### Design and Preparation of Hierarchically Structured DLG

**Fig. 2:**
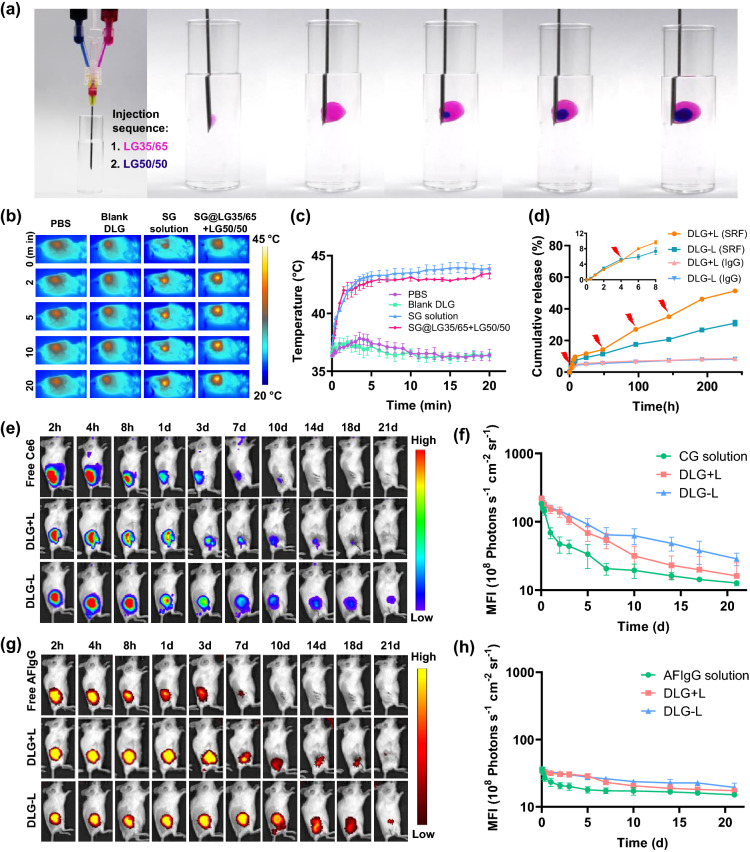
Characterization of the DLG matrix. **a** The formation of a DLG matrix using the dual syringe kit. The outer layer LG35/65 was loaded with Rose Bengal and the inner layer LG50/50 was loaded with Methylene Blue for better illustration. **b** In vivo infrared thermal images of the tumor surgical sites in 4T1-bearing mice irradiated immediately after being treated with PBS, blank DLG, Free SG solution, and LG50/50 + SG@LG35/65. Images were recorded at 0 min, 2 min, 5 min, 10 min, and 20 min after 808 nm laser irradiation. **c** Manually controlled temperature increase in the tumor resection sites on 4T1 tumor-bearing mice, where different formulations were injected, by tuning the NIR laser irradiation for 10 min. **d** The photothermal controlled cumulative release profiles of sorafenib and the long-term sustained cumulative release profiles of IgG from the DLG matrix. Data are shown as means ± SEM (*n* = 3). **e** Fluorescence IVIS images depicting the in vivo retention of Ce6 fluorescence in mice injected with Ce6 solution (Free Ce6), and DLG matrix (CG@lg35/65 + AFIgG@LG50/50) with (DLG + L) or without (DLG-L) the NIR irradiation. **f** Mean fluorescence intensity (MFI) of corresponding fluorescence IVIS images in (**e**). **g** Fluorescence IVIS images depicting the in vivo retention of AFIgG fluorescence in mice injected with AFIgG solution (Free AFIgG), and DLG matrix (CG@lg35/65 + AFIgG@LG50/50) with (DLG + L) or without (DLG-L) the NIR irradiation. H) MFI of corresponding fluorescence IVIS images in (G). Data are shown as means ± SEM (*n* = 3). NIR laser: 808 nm

To prepare the hierarchical DLG matrix, the lipid precursors of the outer layer gel and inner layer gel were loaded into a tailor-made dual syringe kit and injected successively in Fig. 2a. The DLG formed immediately upon injection, with a clear boundary between the outer and inner gel layers (Fig. 2a and Video S1), ascribed to the different gelation properties of the precursors. Sorafenib was firstly adsorbed onto GO to form nanoparticles SG with improved drug-loading efficiency and stability (Figs. S2 and S3). We then employed the Macrosol technique to encapsulate SG in the lipid precursor of the outer layer gel (SG@LG35/65), and aCD47 in the lipid precursor of the inner layer gel (aCD47@LG50/50), respectively, according to a method we developed previously [9], followed by successive injections of the two lipid precursors, using the dual syringe kit, to form the desired DLG SG@LG35/65 + aCD47@LG50/50.

We next examined the photothermal conversion efficiency of the GO-containing DLG matrix using an 808 nm laser. As shown in Fig. S4, the temperature of GO@LG35/65 + LG50/50, which was loaded with GO in the outer layer but kept blank (no aCD47) in the inner layer, increased similarly to a GO solution within an irradiation period of 10 min. In contrast, no obvious change in temperature was observed in a blank PBS solution or a blank DLG matrix (LG35/65 + LG50/50). Within the same irradiation time, the temperature of GO@LG35/65 + LG50/50 increased as the irradiation power went up (Fig. S5), and the photo-induced temperature elevation of the matrix kept constant during five consecutive on/off cycles of irradiation (Fig. S6), exhibiting an excellent photothermal stability similar to the GO solution. The photothermal conversion of the DLG matrix in vivo was also investigated. The infrared thermal images showing the temperature change of 4T1-tumor-bearing mice were recorded (Fig. 2b), after various formulations were injected into the postsurgical tumor resection cavity and irradiated for different time. The constant mild heating for the desired phase transition of the outer layer gel was achieved quickly and maintained by manually tuning the laser power (Fig. 2c). As a comparison, the temperature at the surgical sites of the mice receiving PBS and blank DLG remained unchanged even after 20 min of irradiation, which was consistent with the in vitro results.

The sequential drug delivery characteristics of the hierarchical gel matrix are critical for realizing the therapeutic outcomes of our combined cancer immunotherapy strategy [18]. We evaluated the in vitro drug release profiles of the outer and inner layer gels of the DLG matrix, respectively, using IgG as an alternative of aCD47 (Fig. 2D). The release of sorafenib from SG@LG35/65 was relatively steady without NIR irradiation, and 30% of sorafenib could be released from the outer layer gel within 240 h. When NIR irradiation had been applied at 4, 48, 96, and 144 h, in a pattern similar to that shown in Fig. 2c, significantly enhanced release of sorafenib could be triggered, and over 50% of sorafenib was released from the outer layer gel at 240 h. The release of IgG from the inner layer gel was much more restrained compared to sorafenib, and only less than 10% of IgG could be released from IgG@LG50/50 at 240 h, regardless of the NIR irradiation on the DLG formulation.

To visualize the thermal-responsive sequential release process, we loaded AF 790 goat anti-mouse IgG as an alternative of aCD47 in the inner layer gel precursor (AFIgG@LG50/50) and Chlorine 6-capsulated GO (CG) as an alternative of SG in the outer layer gel precursor (CG@LG35/65). The dye-loaded DLG matrix precursors were injected into the tumor resection cavity, and the fluorescence of labeled IgG and Ce6 was monitored by an in vivo imaging system (IVIS). As shown in Fig. 2e, f, the fluorescence of Ce6 in the DLG matrix showed a prolonged retention in mice than the CG PBS solution, and the fluorescence intensity attenuated more rapidly upon NIR irradiation, exhibiting an effective laser-triggered controlled drug release by the outer layer gel of the DLG matrix. Interestingly, the fluorescence of AFIgG exhibited negligible difference in the injected DLG matrix with or without NIR irradiation, and both groups endowed a long fluorescence retention of over 14 d (Fig. 2g, h), suggesting that the inner gel layer of the DLG matrix enabled a sustained drug release unaffected by external laser irradiation. Collectively, the hierarchical DLG matrix providing hierarchical drug release patterns was proved to be an ideal platform for on-demand sequential drug delivery.

### Programming Sorafenib Release for the Optimized Tumor Microenvironment Modulation

**Fig. 3:**
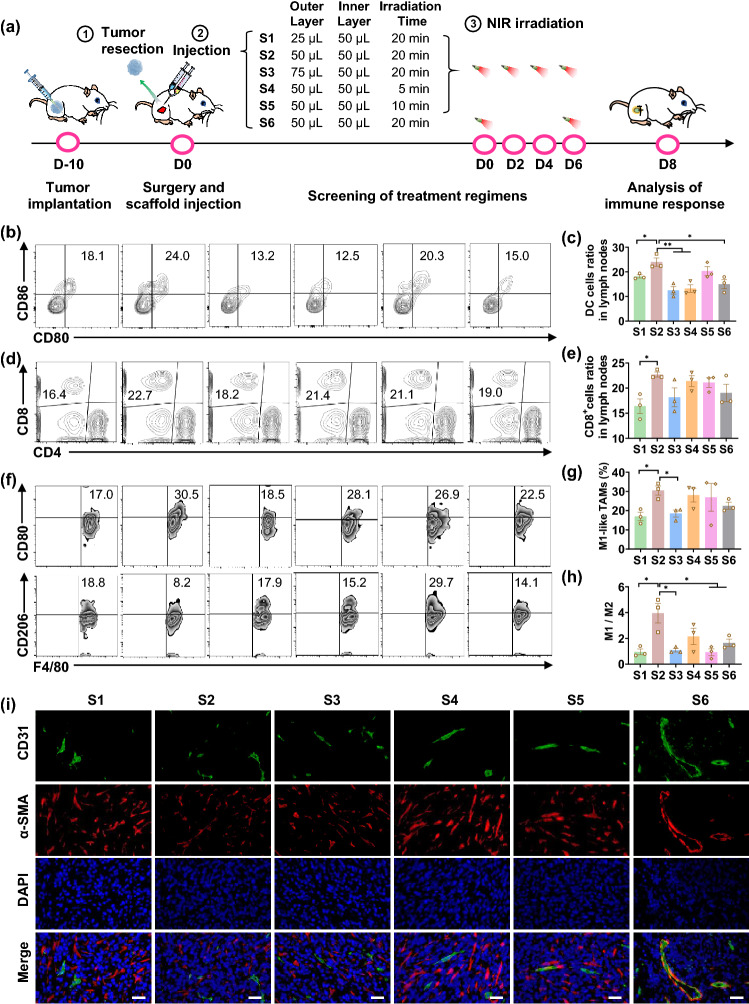
Postsurgical immune responses of different DLG-based treatment strategies on 4T1-tumor-bearing mice. **a** Schematic depiction of the screening process of different treatment strategies. DLG outer layer: SG@LG35/65; DLG inner layer: aCD47@LG50/50; Dose of sorafenib: 50 μg per mouse; Dose of aCD47: 70 μg per mouse. **b**, **c** The representative flow cytometry analysis (**b**) and relative quantification (**c**) of DCs in lymph nodes of tumor resected mice (Gated on CD11^+^ CD80^+^CD86^+^ cells). Data are shown as means ± SEM (*n* = 3). **d**, **e** Representative flow cytometry analysis (**d**) and relative quantification (**e**) of tumor-infiltrating T cells in lymph nodes (gated on CD8^+^ cells). Data are shown as means ± SEM (*n* = 3). **f** Representative flow cytometry analysis of M1-like macrophages (CD80^+^) and M2-like macrophages (CD206^+^) gating on F4/80^+^CD11b^+^ cells. **g** Relative quantification of M1 macrophages (CD80^+^) gating on F4/80^+^CD11b^+^ cells. Data are shown as mean ± SEM (*n* = 3). **h** The ratio of M1/M2 in different groups. Data are shown as mean ± SEM (*n* = 3). **i** Immunofluorescence staining of tumor microvessels in tumor tissue on Day 8 after different treatments. The vascular endothelial cells were stained with the FITC-anti-CD31 antibody (green); the vascular smooth muscle cells were stained with Cy5-anti-α-SMA antibody (red); the cell nucleus was stained with DAPI (blue) (Scale bar: 100 μm). The statistical comparison between groups was performed following the student’s t test (two-tailed). *p < 0.05 and **p < 0.01

A growing number of studies and clinical trials have demonstrated that sorafenib not only triggers the apoptosis of tumor cells by inhibiting the angiogenesis of tumors, but also induces the aggregation of macrophages in TME [26, 43, 44]. In addition, an appropriate dose of sorafenib can impede the polarization of macrophages to M2-like phenotype and reverse immunosuppressive tumor microenvironment (TME), suggesting that the macrophage modulation and antitumor efficacy of sorafenib are dose dependent [26, 27, 45]. Therefore, we posited that the effect of sorafenib on optimizing TME could be facilely optimized by programming the volume ratio of the inner/outer layer gel of the DLG matrix as well as the NIR irradiation frequency and timing. As a proof of concept, we screened a series of DLG matrices (SG@LG35/65 + aCD47@LG50/50) with a fixed inner gel volume and different outer gel volumes, together with a variety of NIR irradiation regimens (Fig. 3a) to approach a maximized antitumor immune response. The doses of aCD47 and sorafenib were set as 70 and 50 μg per mouse, respectively. The study was executed on the surgical beds of randomly grouped 4T1-tumor-bearing mice, in each of which ~ 90% of the tumor was resected when the tumor volume reached approximately 300 mm^3^. The frequencies of immunocytes in lymph nodes and tumor sites of the tested mice were analyzed on Day 8 after surgery. As shown in Fig. 3b, c, the percentage of mature DCs (CD11c^+^CD80^+^CD86^+^) in Group S2 was ~ 24.0%, significantly higher than that in any other group. DCs are key antigen-presenting cells and play an important role in initiating and controlling the innate and adaptive immunity. More mature DCs should induce a stronger systemic antitumor response. As expected, the population of tumor-infiltrating CD8^+^ T cells and CD4^+^ T cells in Group S2 showed the strongest effect on producing antitumor T cell responses among all treatment groups (Figs. 3d, e and S7). Moreover, the proportion of M1-like TAMs (CD80^+^CD11b^+^F4/80^+^) in Group S2 was the highest among all groups, whereas the proportion of M2-like TAMs (CD206^+^CD11b^+^F4/80^+^) in Group S2 was the lowest (Figs. 3f, g and S8). Accordingly, the M1/M2 ratio in Group S2 greatly surpassed all other groups, which was 4.1-fold, 3.6-fold, 2.0-fold, 4.1-fold, and 2.3-fold higher than that in Group S1, S3, S4, S5, and S6, respectively (Fig. 3h). In addition, immunofluorescence staining of tumor microvessels visualized the marked inhibition of angiogenesis of the residual tumor tissue in Group S2 (Fig. 3i). The above results evidenced that regulated release of sorafenib by programming the outer gel volume of DLG matrix as well as the frequency and timing of NIR irradiation could effectively reverse postsurgical immunosuppression, exhibiting great potential to sensitize tumor cells to immune checkpoint blockade therapy. On basis of the above screening, the treatment pattern in Group 2, i.e., a DLG matrix consisting 50 μL of outer layer and 50 μL of inner layer together with a 20 min of NIR irradiation on Days 0, 2, 4, and 6, was selected for further antitumor effect studies.

### In Vivo Antitumor Efficacy of Time-programmed Sequential Delivery of Combined Cancer Immunotherapy via the DLG Matrix

**Fig. 4:**
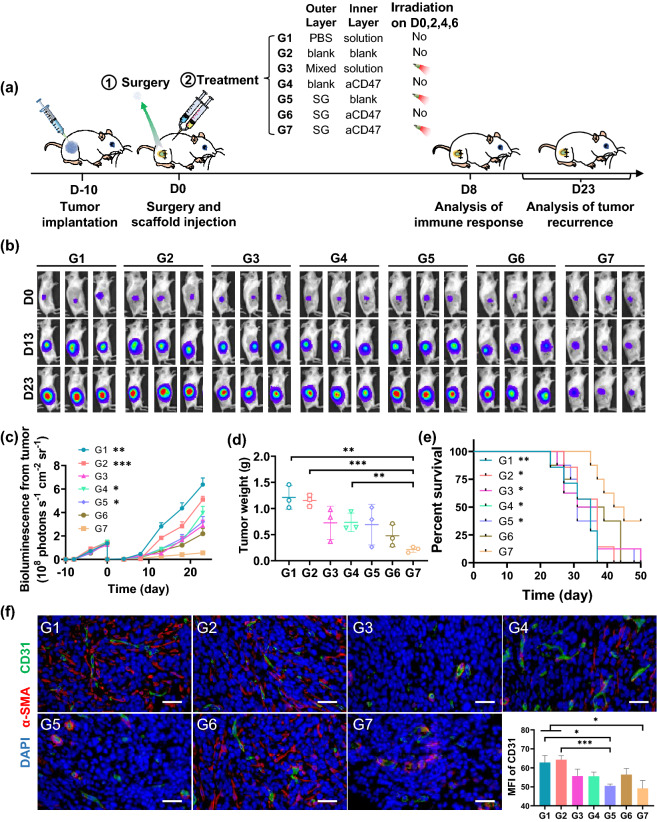
Sequential delivery of combined immunotherapy for inhibiting recurrence of 4T1 carcinoma tumor after surgery. **a** Schematic illustration of the animal experimental design. DLG outer layer: 50 μL; DLG inner layer: 50 μL; Dose of sorafenib: 2.5 mg kg^−1^; Dose of aCD47: 3.5 mg kg^−1^. **b** In vivo bioluminescence imaging of tumor resected mice receiving various treatments (G1–G7) after surgery. Three representative mice in each treatment group are shown. Images associated with Day 0 were taken on the day of surgery. **c** Tumor growth curves with the mean tumor volumes of 4T1 tumor-bearing BALB/c mice model. Data are shown as mean ± SEM (*n* = 5). Data difference is shown comparing to G7. **d** The recurrence tumor weights of different groups obtained from the 4T1-tumor resection mice on Day 23 after treatment. Data are shown as mean ± SEM (*n* = 3). **e** The survival percentages corresponding to the tumor volume of the 4T1-tumor resection mice (*n* = 8). Data difference is shown comparing to G7. **f** Immunofluorescence staining for tumor microvessels in tumor tissue on Day 8 after different treatments. The vascular endothelial cells were stained with the FITC-anti-CD31 antibody (green) and the vascular smooth muscle cells were stained with Cy5-anti-α-SMA antibody (red) and the cell nucleus was stained with DAPI (blue) (Scale bar: 100 μm). The comparison of two groups was followed by student’s t test (two-tailed). *p < 0.05, **p < 0.01, and ***p < 0.001

To evaluate the antitumor efficacy of time-programmed sequential delivery of combined cancer immunotherapy via the above screened treatment regimen, we used an incompletely resected 4T1-tumor mouse model and inspected the inhibition of tumor recurrence after surgery (Fig. 4a). The tumor-bearing mice were randomly grouped and approximately 90% of the tumor was surgically resected when the tumor volume reached ~ 300 mm^3^. The tumor resection cavities were then injected with different formulations before suture, including PBS solution (G1), LG35/65 + LG50/50 (G2), mixed aCD47 and SG solution in PBS (G3), LG35/65 + aCD47@LG50/50 (G4), SG@LG35/65 + LG50/50 (G5), SG@LG35/65 + aCD47@LG50/50 (G6), SG@LG35/65 + aCD47@LG50/50 (G7). The volumes of outer layer gel and inner layer were both set to 50 μL, and the doses of aCD47 and sorafenib were 70 μg and 50 μg per mouse, respectively. After injection, the surgical beds on mice in G3, G5, and G7 were irradiated by an 800 nm laser for 20 min on Days 0, 2, 4, and 6, respectively.

The growth of tumor residues was then monitored by bioluminescence signals from 4T1-luc tumor cells (Fig. 4b). The mice in G7 showed significantly weaker tumor fluorescence (Fig. 4c) than other groups in the whole experimental period (23 d), and the tumor weight on Day 23 in G7 was also the lowest among all treatment groups (Figs. 4d and S9, S10), exhibiting an enhanced inhibition on tumor regrowth by treatment of SG@LG35/65 + aCD47@LG50/50 and NIR irradiation. In addition, the mice receiving this treatment exhibited significantly prolonged survival periods compared to the mice receiving other treatments (Fig. 4e), and the body weights of mice in all groups were not impacted by the treatment (Fig. S11). Moreover, hematoxylin and eosin (H&E) stained images of major organs collected from mice on Day 23, including heart, liver, spleen, lung, and kidney, exhibited minor side effects and inflammation infiltration (Fig. S12), indicating that the time-programmed local delivery of these therapeutics did not induce apparent side effect to mice. Furthermore, the complete blood panel test and serum biochemistry assay illustrated that our injectable double-layer-gel (DLG) preparation has good biocompatibility (Fig. S13).

More importantly, the mice treated with free SG and aCD47 solution and NIR irradiation (G3), or with only aCD47 (G4), showed remarkable tumor suppression compared with the control groups (G1 and G2) within the first several days. However, obvious tumor growths were observed in both G3 and G4 after Day 13, proving that the sequential delivery of SG and aCD47 via the DLG matrix was critical to maintain a long-term tumor inhibition effect, and the prior sorafenib release from the outer layer significantly amplified the antitumor efficacy of long-term released aCD47. In addition, immunofluorescence staining of the microvessels of the recurrent tumors was also examined on Day 8 after different treatments. Figure 4f shows that the signals of CD31 and α-SMA in the vascular endothelial cells in the sorafenib-releasing DLG groups (e.g., G5 and G7) were weaker than those in the other groups. The expression levels of VEGFR2 and PCNA in G7 group were less than in other groups (Fig. S14), verifying the perfect inhibition effect of sorafenib on tumor vessels regeneration and tumor cells proliferation in the recurrent tumors via the designer DLG matrix.

### Immune Responses Induced by the Programed Sequential Delivery of Combined Cancer Immunotherapy

**Fig. 5:**
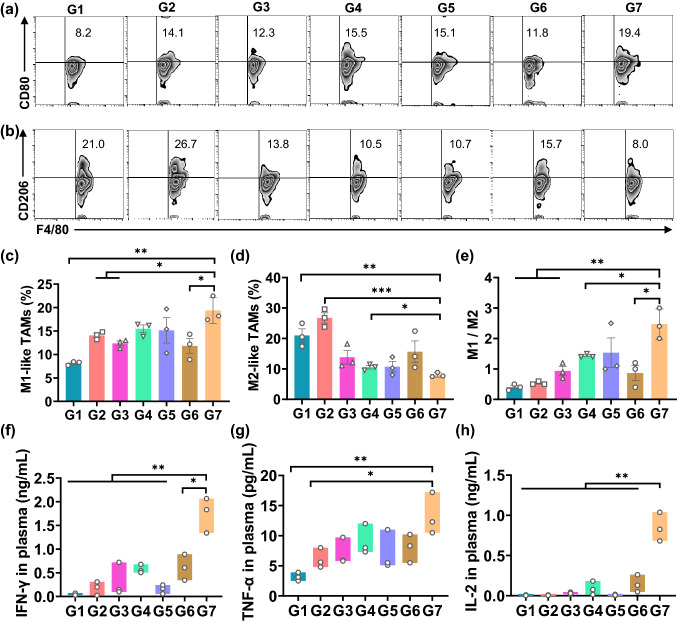
Modulation of TAMs on tumor resected mice induced by DLG matrix-based treatment strategy. G1–G7 are identical to the groups in Fig. 4. **a**, **b** Representative flow cytometric analysis of M1-like TAMs (CD80^+^) and M2-like TAMs (CD206^+^) gating on F4/80^+^CD11b^+^ cells. **c**, **d** Relative quantification of the proportions of M1-like TAMs (CD80^+^) and M2-like TAMs (CD206^+^) gating on F4/80^+^CD11b^+^ cells. Data are shown as mean ± SEM (*n* = 3). **e** The ratio of M1/M2 in different groups. Data are shown as mean ± SEM (*n* = 3). **f–h** Cytokine levels (IFN-γ, TNF-α, IL-2) isolated from the plasma of the mice in different treatment groups on Day 8 after surgery. Data are shown as mean ± SEM (*n* = 3). The statistical comparison between groups was performed following the student’s t test (two-tailed). *p < 0.05 and **p < 0.01

The exceptional inhibition effect on tumor recurrence inspired us to rationale the antitumor immune response triggered by the time-programmed sequential delivery of combined cancer immunotherapy. To validate if the firstly released sorafenib could reeducate TAMs as designed, we investigated the relative proportions of M1-like TAMs and M2-like TAMs at the tumor resection sites using flow cytometry assays. Strikingly, compared with the mice in G1 group, the mice in G7 group showed a significant increase in the relative population of M1-like phenotype (CD80^+^CD11b^+^F4/80^+^) from 8.2% to 19.4% (Fig. 5a, c), along with a large reduction of the relative population of M2-like phenotype (CD206^+^CD11b^+^F4/80^+^) from 21.0% to 8.0% (Fig. 5b, d). The mice receiving other treatments all showed increased portion in M1-like phenotype and decreased portion in M2-like phenotype, but to a much less extent. Therefore, the population ratio of M1/M2 in G7 was significantly higher than that in any other group (Fig. 5e), suggesting that the reeducation/modulation of TAMs was effective and largely dependent on the time-programmed photo-controlled release of sorafenib. The reeducation of TAMs was further confirmed by the secreted cytokines in mice on Day 8 post-injection. We observed upregulated levels of proinflammatory cytokines, which are partially secreted by M1-like TAMs and can activate T lymphocytes and enhance T helper 1 type immunity [46, 47], including interferon-γ (IFN-γ, Fig. 5f), tumor necrosis factor-α (TNF-α, Fig. 5g), interleukin 2 (IL-2, Fig. 5h), and interleukin 6 (IL-6, Fig. S15). On the contrary, the immunosuppressive cytokine interleukin 10 (IL-10) level in plasma, a predominant cytokine secreted by the M2-like phenotype, was significantly downregulated in the mice receiving the designed treatment (G7) compared to the mice in other groups (Fig. S16).

**Fig. 6:**
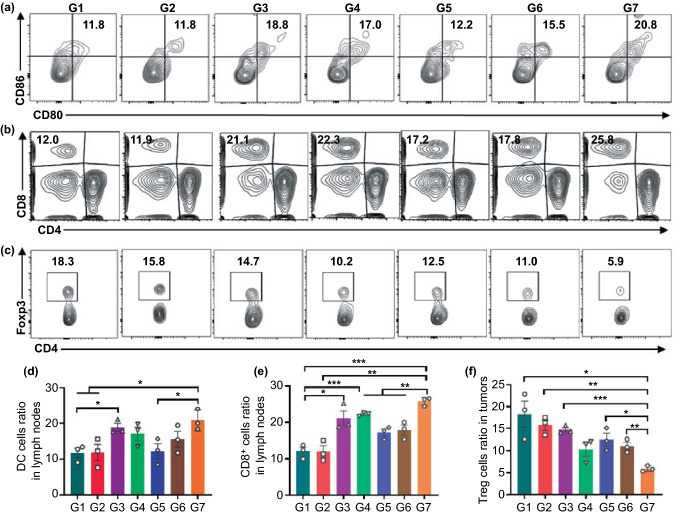
Immune responses on tumor resected mice induced by DLG matrix-based treatment strategy. **a-c** Representative flow cytometric analysis of mature DCs (gated on CD11c^+^ DC cells), tumor-infiltrating of CTLs (gated on CD3^+^ T cells), and CD4^+^Foxp3^+^ Tregs (gated on CD3^+^CD4^+^ cells) from different groups of 4T1 tumor resection mice examined on Day 8 after different treatments. **d-f** The relative quantification of mature DCs, CD8^+^ T cells, and Tregs on Day 8 after different treatments. Data are shown mean ± SEM (*n* = 3). The statistical comparison between groups was performed following the student’s t test (two-tailed). *p < 0.05, **p < 0.01 and ***p < 0.001

We next examined the immune response in lymph nodes and tumor sites of mice on Day 8 after surgery. As expected, the population proportion of mature DCs in lymph nodes of the mice receiving the designed treatment was the highest (~ 20.8%, G7) among all groups (Fig. 6a, d). Correspondingly, the populations of tumor-infiltrating CD8^+^ T cells and CD4^+^ T cells in both lymph nodes and tumor resection sites were higher in G7 than in other groups (Figs. 6b, d and S17–S19). In addition, the DC maturation and CD8^+^ T cells in mice that had received aCD47 treatments (G3 and G4) were also upregulated compared with the blank group (G1), evidencing that CD47 blockade could effectively activate the T cells immune responses and reverse the immunosuppressive postsurgical microenvironment (Fig. 6d, e). Moreover, we examined the level of regulatory T cells (Tregs, CD4^+^CD25^+^Foxp3^+^), which can restrain the antitumor immune effects of cytotoxic T lymphocytes (CTLs) and induce an immunosuppressive microenvironment [48], in the local recurrent tumors by flow cytometry on Day 8 post-injection. As shown in Fig. 6c, f, the frequency of Tregs in the G7 was considerably lower than that in the other groups, indicating that the immunosuppressive microenvironment at tumor resection sites was significantly reversed by the time-programmed sequential delivery of combined immunotherapy.

### Long-Term Immune-Memory Effects

**Fig. 7:**
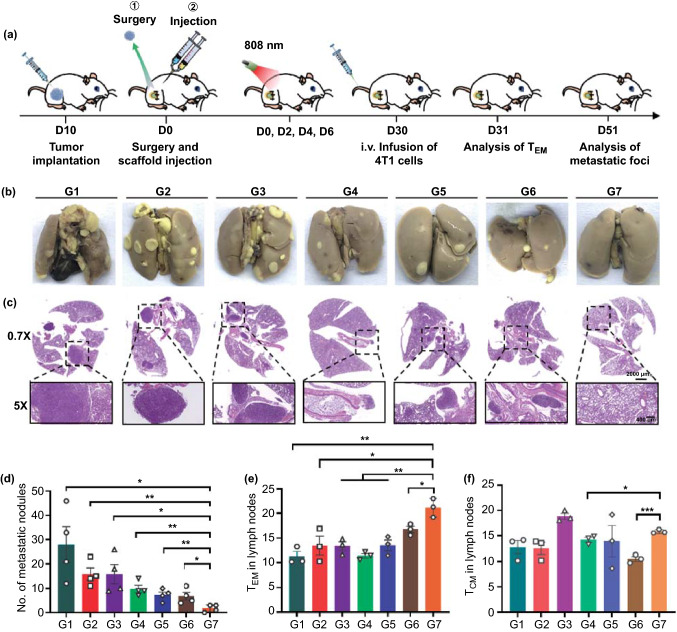
Metastasis prevention via the DLG matrix-enabled sequential delivery strategy. **a** Schematic illustration of the animal experimental design for inhibition of lung metastasis. **b** Representative photographs of lung tissues with tumor metastasis. **c** H&E staining of lung tissues collected from mice in different groups at Day 51 after surgery. Scale bars: 2 mm (top) and 400 μm (bottom). **d** Quantification of pulmonary metastasis nodules in different groups of 4T1 tumor-bearing BALB/c mice. Data are shown as mean ± SEM (*n* = 4). **e**, **f** Proportions of T_EM_ cells and T_CM_ cells in lymph nodes (gated on CD3^+^ CD8^+^ T cells) examined after 1 day post-intravenous infusion of the 4T1 cells. Data are shown as mean ± SEM (*n* = 3). The comparison of two groups was followed by student’s t test (two-tailed). *p < 0.05, **p < 0.01 and ***p < 0.001

We further evaluated whether the local sequential delivery of combined cancer immunotherapy via the DLG matrix could induce systemic immune memory, using a more aggressive whole-body spreading tumor model. In this experiment, the treatment plan kept the same as the above anticancer experiment until Day 30, when all the mice were intravenously injected with 4T1 cancer cells (Fig. 7a). The mice were sacrificed after feeding them for another 21 days and the lung tissues were harvested for metastasis analysis in different groups. As shown in Fig. 7b, c, lung metastatic foci, as well as dense tumor mass on H&E staining of lung sections, were found in all groups except G7, suggesting that our strategy of sequentially delivering combined cancer immunotherapy via the designer DLG matrix was successful in inhibiting the lung metastasis. Additionally, the number of pulmonary metastasis nodules in G7 was remarkably lower than that in all control groups, and it was even 16-fold lower than that in G1 (Fig. 7d). We next carried out a series of memory T cell analysis to have an in-depth understanding of the desirable antitumor immune memory generated by this strategy. In general, effector memory T cells (TEM) are activated immediately by producing cytokines such as IFN-γ, while central memory T cells (TCM) need longer time to produce cytotoxic T cells until being repetitively stimulated by antigens [49–51]. The proportion of TEM in the lymph nodes in G7 was significantly higher than those in any other group (Figs. 7e and S20) on the 1^st^ day after reinjecting 4T1 cells, while an increased population of TCM can be found in G7 compared to other groups except G3 (Figs. 7f and S20). These results further declared that the DLG matrix-based time-programmed sequential delivery strategy was beneficial for the establishment of long-term antitumor immune memory.

## Conclusions

In summary, we have developed a hierarchical DLG matrix, composed of dual SPC/GDO LG layers with different mass ratios, for local time-programmed sequential delivery of combined cancer immunotherapy. Both the in vitro and in vivo drug release studies confirmed that sorafenib could be released on demand from the outer layer upon manually controlled NIR irradiation, followed by a relatively slower release of aCD47 from the inner layer. More interestingly, the volume ratio of the outer/inner layers, as well as the reversible gel-to-sol transition of the outer layer, is programmable to achieve regulated, sequential delivery of various therapeutics for tumor immune microenvironment modulation. As a proof of concept, we screened a series of treatment regimens by modulating the volume of outer layer at a fixed inner layer, as well as the laser irradiation timing and frequency to approach an appropriate release dosage of sorafenib for the optimal antitumor immune responses. We successfully demonstrated that the time-programmed sequential delivery of sorafenib and aCD47 could reeducate TAMs, reverse immunosuppressive TME, and enhance the CD47-blockade efficacy to inhibit the tumor recurrence, when applied to the surgical beds of mice with resected 4T1 tumors. Notably, the local treatment strategy further generated a systemic anticancer immune memory that prevented lung metastasis. This injectable hierarchically structured DLG matrix holds a great potential as a local drug delivery platform to enable time-programmed sequential drug delivery on demand for enhanced therapeutic efficacy of immune-oncology.

## Supplementary Information

Below is the link to the electronic supplementary material.

Supplementary file1 (PDF 945 kb)

Supplementary file2 (MP4 23220 kb)
