# Supplementary material for: Time-Programmed Delivery of Sorafenib and Anti-CD47 Antibody via a Double-Layer-Gel Matrix for Postsurgical Treatment of Breast Cancer
Source: Nanomicro Lett. 2021 Jun 12;13:141. doi: 10.1007/s40820-021-00647-x (PMC8197688; doi:10.1007/s40820-021-00647-x)
Supplement: Supplementary file 1 — Supplementary file1 (PDF 945 kb) [file 40820_2021_647_MOESM1_ESM.pdf]

Supporting Information for

Time-Programmed Delivery of Sorafenib and Anti-CD47 Antibody via a Double-Layer-Gel Matrix for Postsurgical Treatment of Breast Cancer

Liping Huang[1], Yiyi Zhang[1], Yanan Li[2, 4], Fanling Meng[1, 3], Hongyu Li[1], Huimin Zhang[1], Jiasheng Tu[2], Chunmeng Sun[2, *], Liang Luo[1, 3, *]

[1]National Engineering Research Center for Nanomedicine, College of Life Science and Technology, Huazhong University of Science and Technology, Wuhan, 430074, P. R. China

[2]State Key Laboratory of Natural Medicines, NMPA Key Laboratory for Research and Evaluation of Pharmaceutical Preparations and Excipients, and Department of Pharmaceutics, School of Pharmacy, China Pharmaceutical University, 24 Tong Jia Xiang, Nanjing 210009, China

[3]Hubei Key Laboratory of Bioinorganic Chemistry and Materia Medica, School of Chemistry and Chemical Engineering, Huazhong University of Science and Technology, Wuhan 430074, P. R. China

[4]School of Food Science and Pharmaceutical Engineering, Nanjing Normal University, Nanjing 210023, P. R. China

Liping Huang, Yiyi Zhang, and Yanan Li contributed equally to this work.

*Corresponding authors. E-mail: suncm_cpu@hotmail.com (Chunmeng Sun); liangluo@hust.edu.cn (Liang Luo)

S1 Experimental Section

Materials: Soybean phosphatidylcholine (SPC, Lipoid S100) were obtained from Lipoid GmBH (Ludwigshafen, Germany) with a purity of 97.6%. It was used as received and stored at -20 °C. Glycerol dioleate (GDO) was a kind gift from Croda, UK, which contained minimally 95% diglycerides according to the producer. All tool compounds were used as obtained. Sorafenib was purchased from Aladdin. Graphene oxide (GO) was purchased from XFNANO Materials Tech Co.,Ltd. (Nanjing, China). aCD47 (catalog no. BE0270) used *in vivo* purchased from Bio X Cell. AF 790 goat anti-mouse IgG (H&L) (catalog no.115-655-146) was purchased from Jackson ImmunoResear. Anti-CD3-PerCP-Cy5.5 (catalog no. 551163), anti-CD4-FITC (catalog no. 553046), anti-CD8-PE (catalog no. 553032) , anti-CD4-FITC (catalog no.

553046), anti-CD25-APC (catalog no. 557192), and anti-Foxp3-PE (catalog no. 563101), anti-CD11b-FITC (catalog no. 557396), anti-CD11c-FITC (catalog no. 557400), anti-CD80-PE (catalog no. 560016), anti-CD86-APC (catalog no. 553692), anti-CD11b-PE (catalog no. 553692) , anti-F4/80-FICT (catalog no. 553692), anti-CD206-PE (catalog no. 553692) , anti-CD80-APC (catalog no. 553692). anti-LY-6G/LY/6C-PE (catalog no. 553128), anti-CD3-FITC (catalog no. 553065), anti-CD8-PerCP-Cy5.5 (catalog no. 553030), anti-CD62L-APC (catalog no. 562910), and anti-CD44-PE (catalog no. 559250) were purchased from BD Biosciences.

Cell lines: The metastatic murine 4T1-luc breast cancer cell lines were purchased from the American Type Culture Collection (ATCC). The 4T1-luc cells were maintained in Roswell Park Memorial Institute (RPMI) 1640 (Gibco, Invitrogen) medium with 10% FBS, penicillin (100 U mL^{-1}), streptomycin (100 U mL^{-1}), and 1% l-glutamine.

Animals. BALB/c mice (6 to 8 weeks old, 18-20 g) were purchased from the Qinglongshan Farms (Nanjing, China). All animals were bred in the pathogen-free facility with a 12 h light/dark cycle at 20 ± 3 ˚C and had ad libitum access to food and water. Animal protocols were performed under the guidelines for human and responsible use of animals in research set by Huazhong University of Science and Technology and China Pharmaceutical University.

Preparation of the aCD47@LG50/50. The drug-loaded LG samples were fabricated via the 'Macrosol' technique according to the reported previously. Briefly, 100 mg of SPC and 1.4 mg of aCD47 were added into 0.5 mL of pure water. The mixture was mixed sufficiently and lyophilized. The oil phase precursor preparation of LG was prepared via mixing appropriate amounts of lipids (SPC/GDO, 50/50) and ethanol (10%). Finally, 1 mL of the prepared LG precursor was introduced into the lyophilized powder system mentioned above. The non-aqueous mixtures were then mixed on a rolling mixer at room temperature until homogenous lipid solution formulations were produced. The preparation of aCD47@LG50/50 was conducted under sterile conditions.

Preparation of the SG nanoparticles. GO nanoparticles were first prepared by ultrasonic crushing. Sorafenib was dissolved in a solution of PEG-400:ethanol = 1:1, which was then added into the graphene concentrate at a mass ratio of 1:1 and mixed overnight at room temperature. Finally, the unloaded drug is removed by centrifugation. The loading contents of sorafenib as weight percentages in GO were calculated using UPLC on the basis of the calibration curves.

Preparation of the SG@LG35/65. The solution of sorafenib-loaded GO was lyophilized. The preparation of LG was prepared via mixing appropriate amounts of lipids (SPC/GDO, 35/65) and ethanol (5%). The non-aqueous mixtures were then mixed on a rolling mixer at room temperature until homogenous lipid solution formulations were produced. The LG preparation was done under sterile conditions.

Characterization of SG nanoparticles. The hydrodynamic diameter of SRF@GO

was determined using DLS (Nano ZS90, Malvern Instrument).

***In vitro* drug release study.** The DLG formed by 100 µL SG@LG35/65 and 100 µL IgG@LG50/50 (IgG, 1.4 mg·mL^{-1}; Sorafenib, 1.0 mg·mL^{-1}) was added into each of the 6 vials. The samples were divided into two groups ("with light" group and "without light" group) and incubated on an shaker at 37 °C. And 3.0 mL PBS (pH 7.4) was used as the drug release media. At the desired interval, all the media of the samples was removed and stored at -20 °C for further analysis and another 1.5 mL of fresh media was then added to each vial. Each sample of the "with light" group was irradiated with an 808 nm laser at a power density of 1.0 W cm^{-2} for 20 min at 4 h, 2 d, 4 d and 6 d after sampling. The released amount of IgG was measured using bicinchoninic acid (BCA) assays and diluted free IgG as a standard curve. The released amount of sorafenib was measured by UPLC (Agilent).

***In vitro* photothermal effect.** To investigate the photothermal stability of GO loaded LG, 100 µL of GO@LG35/65 (0.5 mg GO) and GO@LG35/65+LG50/50 each was placed in a 2 mL tube respectively. All the tubes were irradiated for five cycles with an 808 nm laser on and off at a power density of 1.0 W cm^{-2}. The heating and cooling curves were recorded by an infrared thermal camera (FLIR E50, USA). And free GO dissolved in pure water was used as control treated in the same conditions. The double gel was also irradiated with an 808 nm laser for 10 min at the power density of 0.8 W·cm^{-2} and 1.2 W·cm^{-2}. In addition, free GO solution, PBS and blank LG placed in a 2 mL tube were also irradiated with an 808 nm laser for 10 min at a power density of 1.0 W cm^{-2}.

***In vivo* drug release study.** To evaluate the *in vivo* drug release of Alexa Fluor® 790 AffiniPure Goat Anti-Mouse IgG (an alternative model fluorescent antibody for aCD47) and Ce6, the designated DLG matrix was injected into the surgical bed of each tumor-resected mouse. For the control, the fluorescent molecule solutions were administered into the incision sites. Each mouse in irradiation-receiving groups was then irradiated with an 808 nm laser for 20 min for four times post-surgery. Fluorescence images were obtained at the desired interval and analyzed by a Living Imaging software.

***In vivo* photothermal effect.** After the surgery, LG50/50+50LG35/65, LG50/40+ SG@LG35/65 and free aCD47/SG solution was injected directly into the tumor area of the corresonding mouse. The tumor area was then irradiated with an 808 nm laser at an appropriate power density for 20 min. The thermal image of the whole mouse was recorded by the infrared thermal camera at at 2 h, 2 d, 4 d and 6 d post injection. PBS was used as control treated in the same conditions.

***In vivo* tumor models and treatment.** To evaluate the therapeutic efficiency of a DLG matrix for synergistic cancer therapy, the antitumor study was performed using a 4T1 tumor model. For the tumor inoculation, 4T1-Luc cells (1 × 10^{6}) suspended in PBS were subcutaneously injected into the right flank of each female BALB/c mouse. Mice were randomly divided into six groups, and surgery was performed when tumor reached ~300 mm^{3}. While mice were kept under anesthesia at 2% isoflurane, the

tumor was resected, leaving ~10% residual tumor to mimic residual micro tumors after surgery. Immediately after surgery, the tumor resection cavities were then injected with different formulations, including PBS solution (G1), LG35/65+LG50/50 (G2), mixed aCD47 and SG solution in PBS (G3), LG35/65+aCD47@LG50/50 (G4), SG@LG35/65+LG50/50 (G5), SG@LG35/65+aCD47@LG50/50 (G6), SG@LG35/65+aCD47@LG50/50 (G7). The volumes of outer layer gel and inner layer were both set to 50 μL, and the dose was 70 μg per mouse for aCD47 and 50 μg per mouse for sorafenib. In control experiments, therapy was administered in PBS solution and free aCD47/SG solution at the site of surgery. And the each mouse of light group was then irradiated with an 808 nm laser at an appropriate power density for keeping the temperature below 45 °C for 20 min at 0 d, 2 d, 4 d and 6 d. In the following days, mice were monitored for local tumor recurrence by an *in vivo* imaging system. After 23 days, three mice in each group were sacrificed. The recurrent tumors were taken out to weigh and take photos. The survival time and body weights of the remaining mice were continued to be recorded until day 60 post tumor resection. Finally, all the major organs (i.e., heart, liver, lung and kidney) were collected with the examination by H&E staining.

Lung metastasis analysis. In the lung metastasis experiment, while the treatment plan was not changed and the recurrent tumor on each mouse was removed by surgery on day 30, mice were then intravenously injected with 4T1 cells. After feeding the mice for another 21 days, mice were sacrificed to obtain their lungs and fix in Bouin’s solution. Tumor metastasis sites subsequently appeared as yellow nodules on the surface of lungs and were counted by visual observation. The establishment of 4T1 tumors in the lung was also examined by H&E staining.

Cytokine detection. Serum samples were isolated from mice on day 8 after various treatments and diluted for analysis. Tumor necrosis factor (TNF-α), interferon gamma (IFN-γ), and interleukin 6 (IL-6) were analyzed with ELISA kits according to vendors’ instructions (KeyGEN Biotech, China).

Analysis of infiltrating immune cells of different groups. On Day 8 post surgery, mice were sacrificed, and the recurrent tumors and lymph nodes were surgically resected from mice in different groups. Then lymphocytes in lymph nodes and infiltrating lymphocytes in tumors were obtained after several operation. The collected lymphocytes were incubated with anti-CD3-PerCP-Cy5.5, anti-CD4-FITC, and anti-CD8-PE antibodies according to the standard protocols to determine the content of $CD4^+$ or $CD8^+$ T cells in the tumors and spleens using a flow cytometry. The regulatory T cells was examined by staining the lymphocytes with anti-CD4-FITC (catalog no. 553046), anti-CD25-APC (catalog no. 557192), and anti-Foxp3-PE (catalog no. 563101) antibodies according to the standard protocols. The macrophage was examined by staining the lymphocytes with anti-CD11b-PE, anti-F4/80-FICT, anti-CD206-PE, and anti-CD80-APC antibodies according to the standard protocols. For DC maturation examination *in vivo*, the inguinal lymph nodes were harvested to collect lymphocytes. The frequency of matured DCs in the lymph nodes was then examined by flow cytometry (BD FASCVerse, USA) after immunofluorescence

staining with anti-CD11c-FITC, anti-CD80-PE, and anti-CD86-APC antibodies according to the procedure of the manufacturer. For analysis of memory T cells, bone marrows harvested from mice after various treatment were stained with anti-CD3-FITC, anti-CD8-PerCP-Cy5.5, anti-CD62L-APC, and anti-CD44-PE antibodies according to the manufacturer. The single-cell suspension from lymph nodes was prepared using the same protocol to that of tumor tissues [1]. Central memory T cells (T_{CM}) and effector memory T cells (T_{EM}) were $CD3^+CD8^+CD62L^+CD44^+$ and $CD3^+CD8^+CD62L^-CD44^+$, respectively. All these antibodies used in our experiments were diluted by ~200 times.

Immunofluorescence staining. Tumors were collected from the mice and snap-frozen in optimal cutting temperature medium. Tumor sections were cut using a cryotome, mounted on slides and stained with different primary antibodies: CD31 (Abcam, cat. no. ab183685), α-SMA (Abcam, cat. no. ab22378) and DAPI (Abcam, cat. no. ab100790) overnight at 4 °C following the manufacturer's instructions. Following the addition of fluorescently labelled secondary antibodies, including goat anti-rat IgG (H + L; Thermo Fisher Scientific, cat. no. A18866) and goat anti-rabbit IgG (H + L; Thermo Fisher Scientific, cat. no. A32733), the slides were analyzed with a confocal microscope (Zeiss LSM 710). All antibodies used in the experiments were diluted ~200 times.

Statistical analysis. All the analysis data are given as mean ± SEM. The results were analyzed by the Student's t-test between two groups. *$P < 0.05$ was considered significant. **$P < 0.01$ and ***$P < 0.001$ were highly significant compared to corresponding control.

S2 Supplementary Tables and Figures

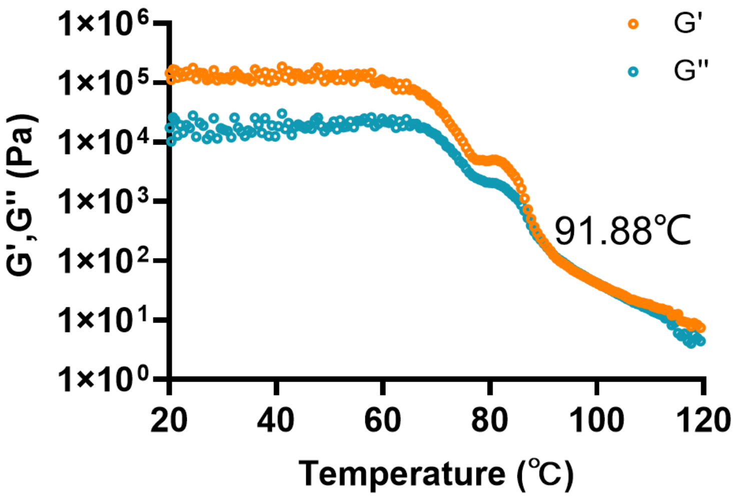

Fig. S1 Temperature-responsive storage (G′) and loss (G″) modulus changes of the LG with an SPC/GDO mass ratio of 50/50

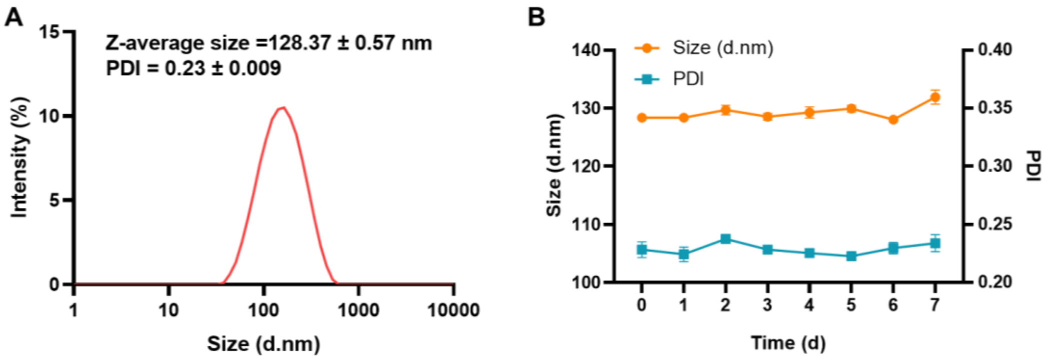

Fig. S2 Size distribution and stability of GO

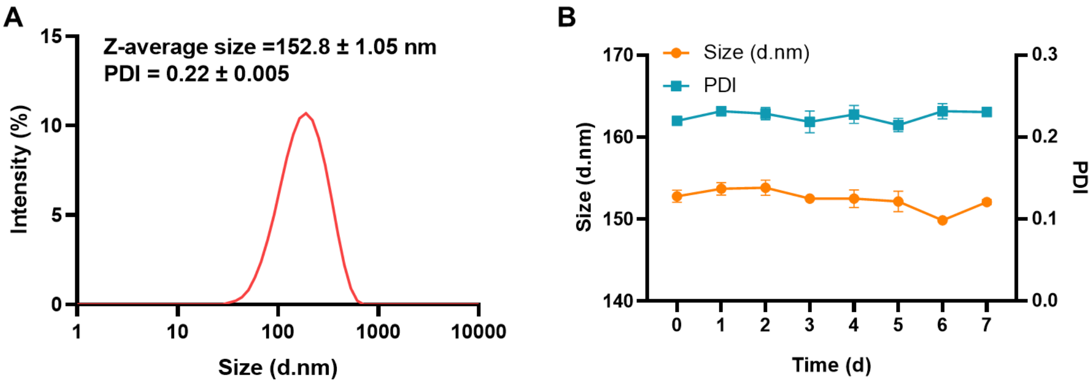

Fig. S3 Size distribution and stability of SG

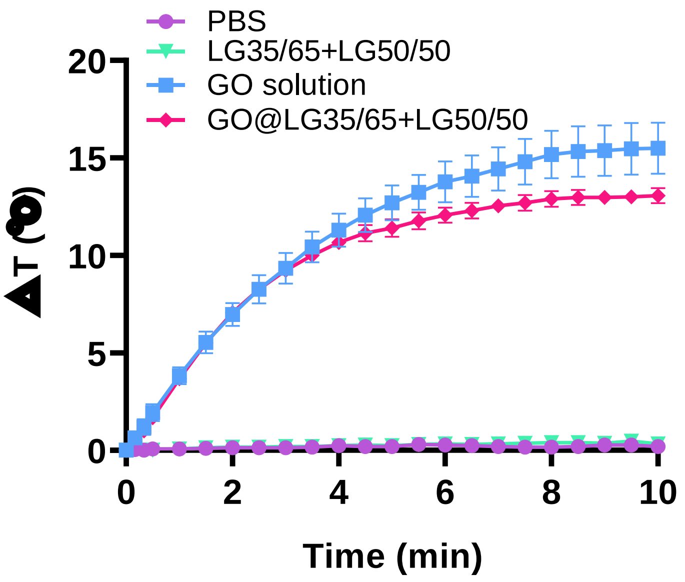

Fig. S4 Temperature increase curves of PBS, LG35/65+LG50/50, GO solution and GO@LG35/65+LG50/50 with the NIR laser irradiation for 10 min *in vitro*

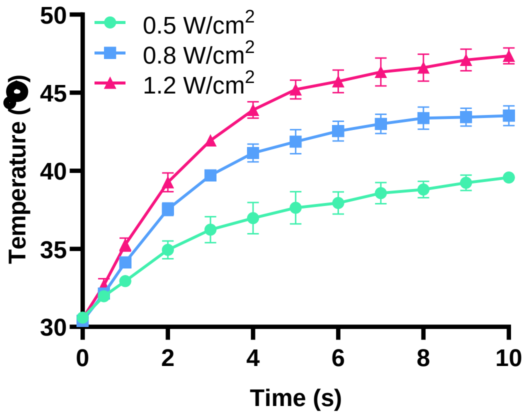

Fig. S5 Temperature increase curves of GO@LG35/65+LG50/50 with the different laser irradiation power for 10 min *in vitro*

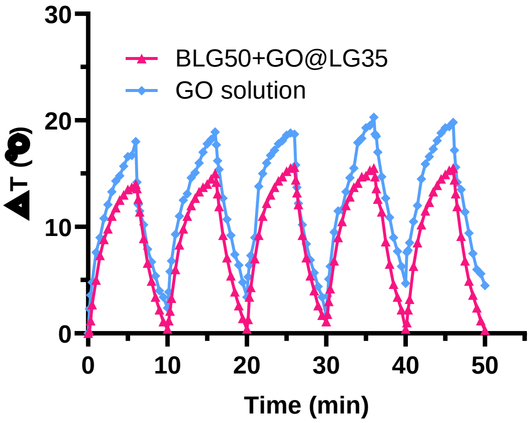

Fig. S6 Heating and cooling curves of GO solution and GO@LG35/65+LG50/50 for five cycles by turning on and off laser

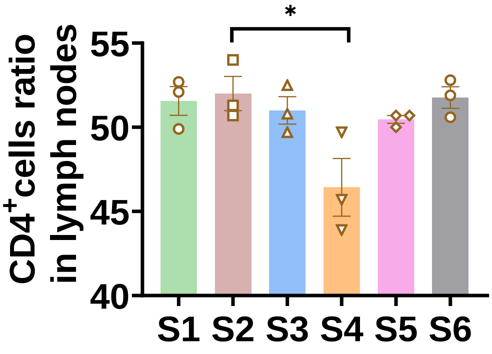

Fig. S7 Quantification results of $CD4^+$ T cells in lymph nodes (gated on $CD3^+$ cells). Data are shown as mean ± SEM (n = 3). The statistical comparison between groups was performed following the student's t-test (two-tailed). *$p < 0.05$, **$p < 0.01$ and ***$p < 0.001$

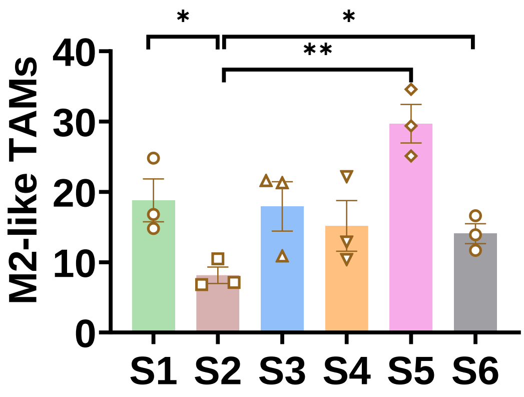

Fig. S8 Relative quantification of M2 macrophages ($CD206^+$) gating on $F4/80^+CD11b^+$ cells. Data are shown as mean ± SEM (n = 3). The statistical comparison between groups was performed following the student's t-test (two-tailed). *p < 0.05, **p < 0.01 and ***p < 0.001

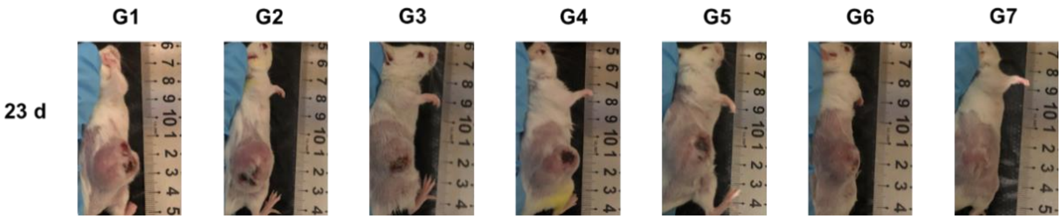

Fig. S9 Representative mice pictures with recurrence tumors on Day 23 after surgery

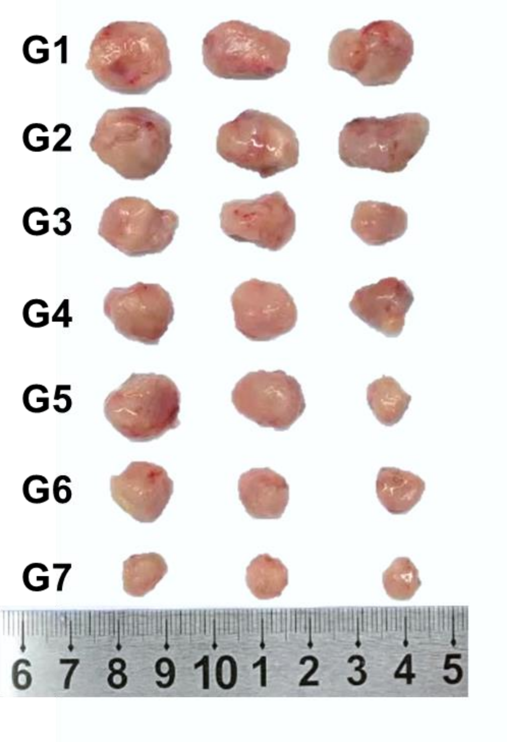

Fig. S10 Photograph of recurrent tumors collected from the mice treated in various groups on Day 23 after surgery

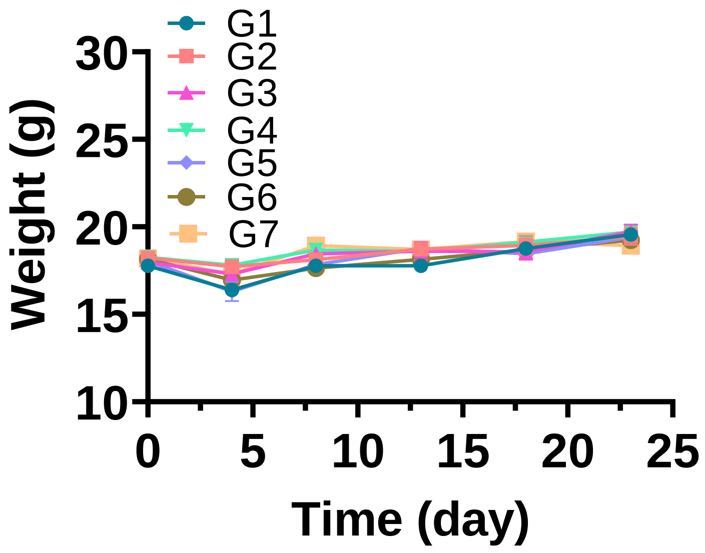

Fig. S11 Body weight change curve of 4T1-luc tumor resection mice. Data are shown as mean ± SEM (n = 5)

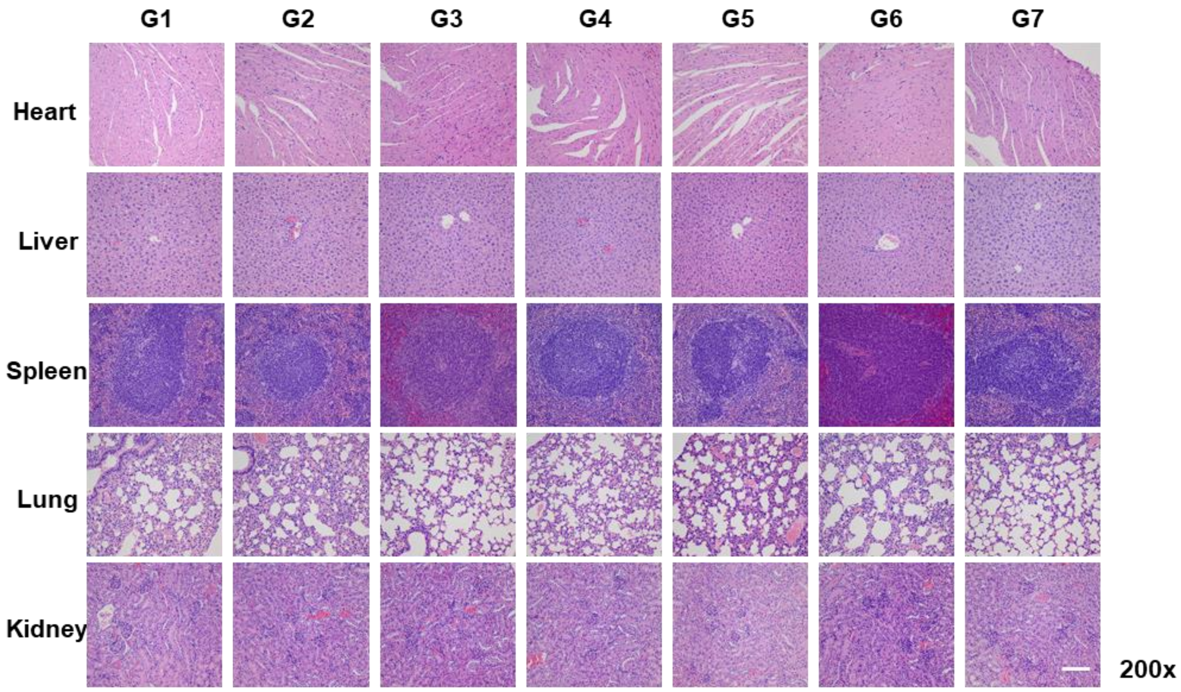

Fig. S12 Histopathological images of the main organs, containing heart, liver, spleen, lung, and kidney obtained from the 4T1-luc tumor resection mice. The images were measured at a magnification of 200× (scale bar: 200 μm)

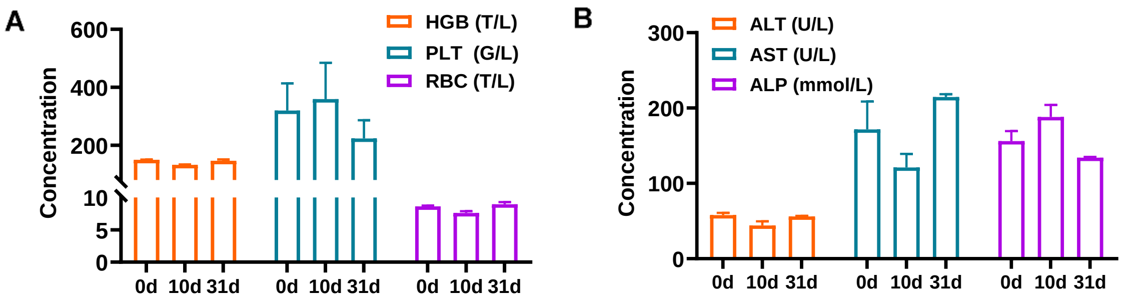

Fig S13 Blood panel test **A**) and serum biochemistry assay **B**) of mice at different time after receiving DLG administration

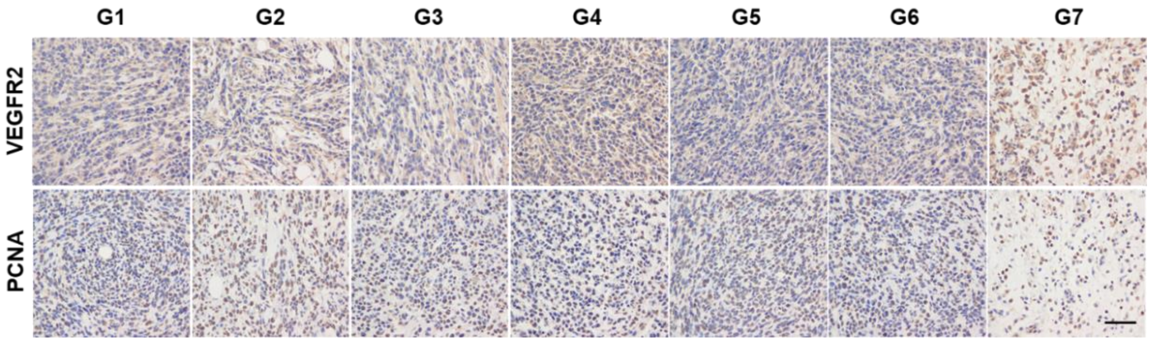

Fig. S14 Immunostaining for VEGFR2 and PCNA were performed in the recurrence tumors. VEGFR2, vascular endothelial growth factor receptor-2. PCNA, proliferating cell nuclear antigen. The images were measured at a magnification of 400×(scale bar: 100 μm)

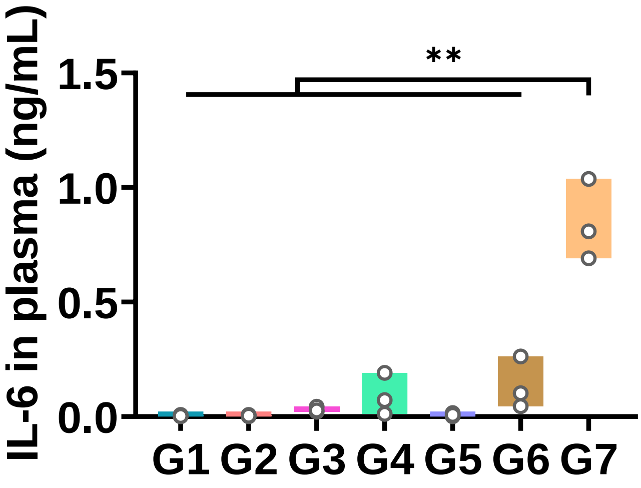

Fig. S15 Levels of IL-6 in the serum from mice isolated 8 days after different treatments. Data are shown as mean ± SEM (n = 3). The statistical comparison between groups was performed following the student's t-test (two-tailed). *p < 0.05, **p < 0.01 and ***p < 0.001

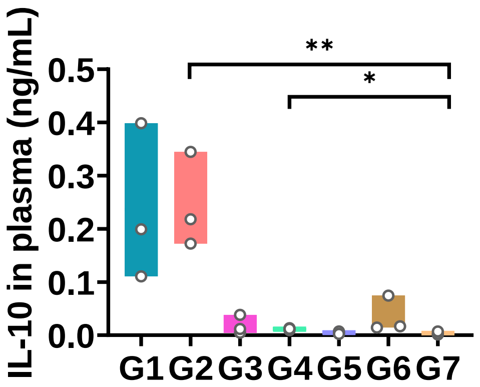

Fig. S16 Levels of IL-10 in the serum from mice isolated 8 days after different treatments. Data are presented as mean ± SEM (n = 3). The statistical comparison between groups was performed following the student's t-test (two-tailed). *p < 0.05, **p < 0.01 and ***p < 0.001

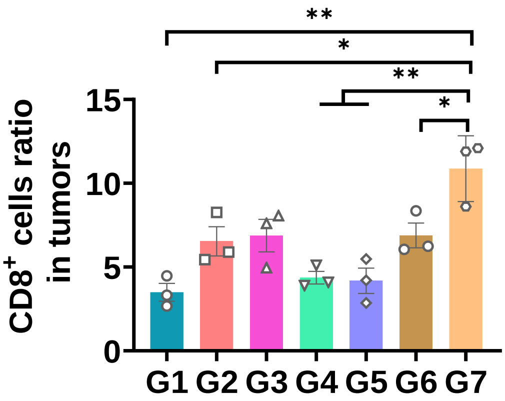

Fig. S17 Quantification results of $CD8^+$ T cells in recurrent tumors (gated on $CD3^+$ cells) on day 8 after surgery. Data are shown as mean ± SEM (n = 3). The statistical comparison between groups was performed following the student's t-test (two-tailed). *p < 0.05 and **p < 0.01

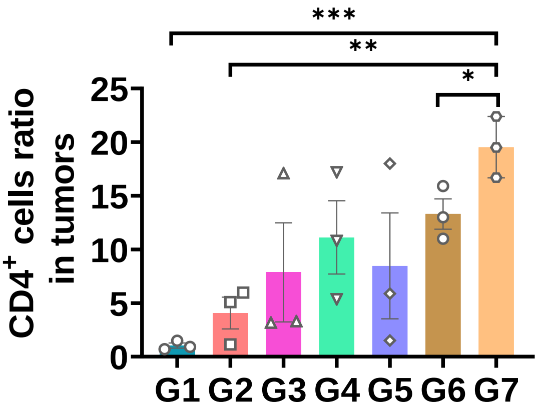

Fig. S18 Quantification results of $CD4^+$ T cells in recurrent tumors (gated on $CD3^+$ cells) on day 8 after surgery. Data are shown as mean ± SEM (n = 3). The statistical comparison between groups was performed following the student's t-test (two-tailed). *p < 0.05, **p < 0.01 and ***p < 0.001

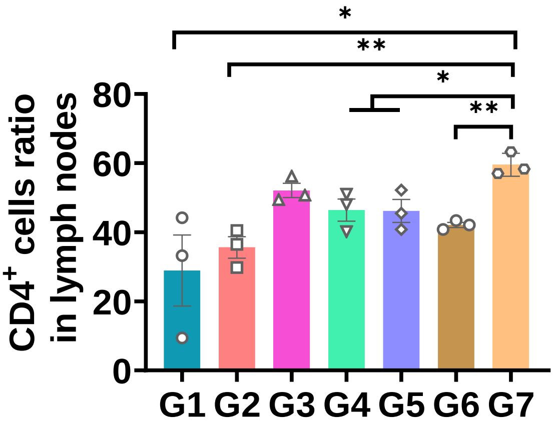

Fig. S19 Quantification results of CD4$^+$ T cells in lymph nodes (gated on CD3$^+$ cells) on day 8 after surgery. Data are shown as mean ± SEM (n = 3). The statistical comparison between groups was performed following the student's t-test (two-tailed). *$p < 0.05$ and **$p < 0.01$

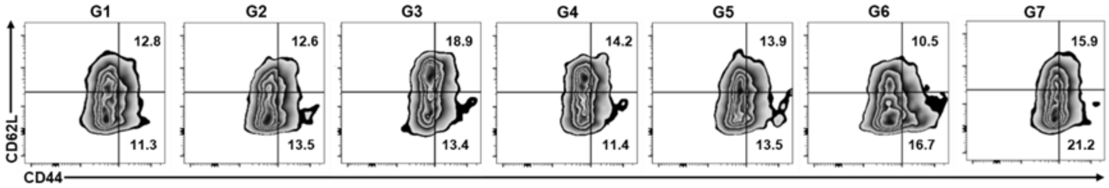

Fig S20 Representative flow cytometric analysis of T$_{EM}$ cells (CD44$^+$) and T$_{CM}$ cells (CD62L$^+$) gating on CD3$^+$ CD8$^+$ T cells

Supplementary Reference

[S1] K. Takahashi, A. Kenji, T. Norihiro, K. Eisaku et al., Morphological Interactions of Interdigitating Dendritic Cells with B and T Cells in Human Mesenteric Lymph Nodes. Am. J. Psychol. **159**, 131-138 (2001). https://doi.org/10.1016/S0002-9440(10)61680-X
